# Analysis of CacyBP/SIP, ERK1/2, and p38 Expression in Low‐ and High‐Grade Papillary Urothelial Carcinoma

**DOI:** 10.1002/cam4.71649

**Published:** 2026-02-15

**Authors:** Natalia Domian, Grzegorz Młynarczyk, Irena Kasacka

**Affiliations:** ^1^ Department of Histology and Cytophysiology Medical University of Białystok Białystok Poland; ^2^ Department of Urology Medical University of Białystok Białystok Poland

**Keywords:** bladder cancer, CacyBP/SIP, ERK1/2, p38, papillary urothelial carcinoma

## Abstract

**Background:**

Papillary urothelial carcinoma (transitional cell carcinoma) is one of the most common malignant tumors of the urinary tract. Urothelial carcinomas are classified into muscle‐invasive and non‐muscle‐invasive types. Among the latter, papillary urothelial carcinoma is further categorized into low‐grade and high‐grade forms. The aggressiveness of bladder cancer depends on the stage and grade of the disease. The CacyBP/SIP protein and MAP kinases play key roles in various cellular processes and signaling pathways that determine cell survival or death. This study aimed to assess the expression of CacyBP/SIP, ERK1/2, and p38 in low‐ and high‐grade papillary urothelial carcinoma using immunohistochemical and molecular analyses.

**Materials and Methods:**

Tissue samples were obtained from 20 patients with high‐grade urothelial carcinoma and 20 patients with low‐grade urothelial carcinoma. Adjacent non‐cancerous tissues served as comparative controls. Immunohistochemistry and qRT‐PCR were used to evaluate the expression of CacyBP/SIP, ERK1/2, and p38.

**Results:**

The strongest expression of the CacyBP/SIP gene was observed in tumor tissues with high malignant potential, predominantly in the nuclear compartment. Similarly, p38 kinase expression was elevated, whereas ERK1/2 expression was reduced in bladder tumor tissues compared to adjacent normal bladder tissues.

**Conclusions:**

These findings suggest that CacyBP/SIP may play a critical role in urothelial carcinoma progression by modulating ERK1/2 and p38 kinase activity.

## 1 Background

Bladder cancer accounts for approximately 3% of all malignancies and is the second most common malignant tumor of the urinary system. It is responsible for 2.1% of cancer‐related deaths, making it the 13th leading cause of cancer‐related mortality worldwide. An increase in the incidence of bladder cancer has been observed in recent decades, particularly in industrialized countries [1, 2, 3, 4]. The most frequently diagnosed type is urothelial carcinoma, which accounts for 90% of cases and originates from the transitional epithelium. Urothelial carcinomas are classified as muscle‐invasive and non‐muscle‐invasive. Among the latter, papillary urothelial carcinoma is further categorized into low‐grade (LG‐UC) and high‐grade (HG‐UC) forms. The aggressiveness of bladder cancer depends on the stage and grade of the disease; high‐grade (poorly differentiated) tumors have a greater potential for progression and metastasis compared to low‐grade (well‐differentiated) tumors [5, 6, 7].

CacyBP/SIP is a multifunctional protein containing multiple domains. It is expressed in a variety of mammalian cells and tissues. It has been shown that the CacyBP/SIP protein in the cytoplasmic compartment, and under the influence of various factors, can translocate to the cell nucleus or the perinuclear area. In recent years, scientists have investigated the involvement of CacyBP/SIP in the occurrence and development of various cancers [8, 9, 10, 11, 12, 13, 14]. Despite the fact that several reports have been published on the implication of CacyBP/SIP in cell proliferation and tumor progression, its mechanism of action is not fully known.

MAP kinases are involved in important processes, including regulating the activity of many proteins, transcription factors, and enzymes. These include ERK1/2 (a kinase regulated by extracellular signaling), p38, and JNK (c‐Jun N‐terminal kinase). ERK1 and ERK2 are related protein‐serine/threonine kinases that participate in the Ras–Raf–MEK–ERK signal transduction cascade, which is involved in the regulation of a wide variety of cellular processes, including survival, adhesion, migration, cell cycle progression, proliferation, differentiation, and transcription [15, 16]. The activity of the Ras–Raf–MEK–ERK cascade has been shown to be increased in approximately one‐third of all human cancers, and inhibition of components of this cascade by targeted inhibitors is an important anti‐cancer strategy [17]. CacyBP/SIP has been shown to be able to bind to and dephosphorylate ERK1/2 kinases. CacyBP/SIP phosphatase activity toward ERK1/2 was found in neuroblastoma NB2a cells, and overexpression of CacyBP/SIP correlated with a decrease in the amount of phosphorylated ERK1/2 in the nuclear fraction [18].

The p38‐MAPK signaling pathway, crucial for cancer cells, enables them to sense and respond to different environmental cues, making it an appealing target for potential anticancer treatments [19, 20]. Growing evidence suggests that p38 signaling plays a dual role in a variety of malignancies, where it can both inhibit and enhance tumor growth, metastasis, and chemoresistance. This dual role of the p38 protein, as well as its activity under various, not fully explained conditions, constitutes a significant obstacle to the development of effective anticancer drugs [21, 22].

The aim of this study was to evaluate the expression levels and immunoreactivity of CacyBP/SIP, ERK1/2, and p38 in low‐ and high‐grade papillary urothelial carcinoma. In addition, the study aimed to compare these findings between tumor tissues and adjacent non‐cancerous tissues to determine potential differences in protein localization and signaling activity. Understanding these molecular alterations may provide insights into the role of CacyBP/SIP and MAP kinases in bladder cancer progression and their potential as prognostic markers or therapeutic targets.

## 2 Materials and Methods

The research material consisted of tissues collected from patients after removal of a urinary bladder tumor at the Department of Urology of the Medical University of Bialystok. The study was approved by the Bioethics Committee of the Medical University of Bialystok. The code for our study is APK.002.109.2023. All studies were performed in accordance with relevant guidelines/regulations. The research was conducted in accordance with the Declaration of Helsinki regarding research involving patients.

The study included patients after transurethral resection of the bladder tumor or after radical cystectomy. The material for testing was collected directly from the tumor in the operating theater. Tissue fragments for immunohistochemistry were fixed in 10% buffered formalin immediately after collection. The Real Time PCR material was immediately placed in RNAlater solution (AM7024 Thermo Fischer) and frozen at −80°C. The study groups consisted of 20 patients with a histopathological diagnosis of high‐grade papillary urothelial carcinoma and 20 low‐grade patients (Table 1). The comparative material consisted of tissues adjacent to the tumor, without microscopic histopathological changes, collected from the same patients.

**TABLE 1:** Patient characteristics/bladder cancer.

	Low grade bladder cancer (non‐muscle invasive)	High grade bladder cancer (muscle invasive)
Average age	62.1	66.4
♀	12	4
♂	8	16
Previous treatment > neadjuvant chemotherapy	0	6
Staging	pTaN0M0—all patients	pT2N0M0—all patients
Average BMI	28.96	29.14
Metastasis	0	0
Arterial hypertension	6	7
Diabetes type 2	2	1
Atrial fibrilation	2	1
Hyperthyroidism	—	1

### 2.1 Identification of CacyBP/SIP, ERK1/2 and p38 by Immunohistochemistry

Immunohistochemical staining was performed according to the following procedure [23]. The tissue samples were first fixed in 10% formalin to preserve their structure. The solid tissue samples underwent dehydration using a series of alcohol solutions. After dehydration, the tissue samples were cleared using xylene. Dehydrated and cleared tissue samples were immersed in molten paraffin wax. Infiltrated tissue samples were placed in molds filled with molten paraffin wax, oriented appropriately, and allowed to solidify. Once the paraffin had solidified, the tissue block was trimmed to remove excess paraffin and then mounted onto a microtome. Thin sections of tissue, typically around 4 μm thick, were cut from the block using a sharp blade. The thin sections of tissue were transferred to glass slides and dried thoroughly. Sections were deparaffinized and rehydrated. Diluted primary antibodies directed against CacyBP/SIP (ab190950 Abcam, 1:600), ERK1/2 (44‐680G Invitrogen, 1:100), and p38 (44‐684G Invitrogen, 1:100) were used for the study. The sections were incubated at 125°C in Target Retrieval Solution Citrate pH = 6.0 antigen unmasking buffer (S2369 DAKO Cytomation), then the endogenous peroxidase was blocked with 0.3% hydrogen peroxide for 10 min. Diluted primary antibodies were spotted on the sections and incubated overnight at 4°C. After incubation, a secondary antibody labeled with horseradish peroxidase (DAKO REAL EnVisionTM Detection System K 5007 DAKO Cytomation) was used for 1 h. In order to visualize the formed antigen–antibody complex, the DAB chromogen was used. Cell nuclei were stained with hematoxyclin QS (H‐3404, Vector Laboratories). Each staining step was preceded by thorough rinsing of the sections in Wash Buffer (S3006 DAKO Cytomation). Specificity tests performed for the CacyBP/SIP, p‐ERK1/2, and p‐p38 antibody included negative control, where the primary antibodies were omitted, only antibody diluent was used, and a positive control was prepared with specific tissue as it was recommended by the manufacturer. Histological preparations were evaluated using an Olympus BX43 light microscope (Olympus 114 Corp.) with an Olympus DP12 digital camera (Olympus 114 Corp.) and documented. Each obtained digital image of the bladder cancer and normal tissue was morphometrically evaluated using NIS Elements AR 3.10 Nikon software for microscopic image analysis. The intensity of the immunohistochemical reaction for all the antibodies used in the study was measured on each image analyzed and determined using a gray scale level 0 to 256, where the value of the completely white or bright pixel is 0, while the completely black pixel is 256.

The positive control included tissue known to express the test antigen, performed in a manner analogous to the test tissue. In this control, only structures expected to express antigen tested positive. The remaining cells and stromal elements were negative. In the negative control, we replaced the primary antibody with a diluent or non‐specific antibody of the same isotope, the same species and the same concentration (depending on whether it was a commercial antibody or one from the laboratory where it was produced, e.g., against CacyBP/SIP). There was no specific staining in the negative control.

### 2.2 Real‐Time PCR

Sections of bladder cancer (fresh frozen tissue samples) and tumor‐adjacent normal bladder tissue were collected from each patient and placed in Eppendorf tubes in RNA‐later solution (AM7021, Invitrogen). The collected material was stored at −80°C. Total RNA was isolated using the Machery‐Nagel NucleoSpin RNA isolation kit. The amount and control of the isolated RNA (RNA quality assessment based on 260/280 values) were determined using a NanoDrop 2000 spectrophotometer (ThermoScientific). Total RNA was reverse transcribed into cDNA using the iScript Advanced cDNA Synthesis Kit for RT‐qPCR from BIO‐RAD. cDNA synthesis was performed using a thermal cycler: model SureCycler 8800, Aligent Technologies. The reverse transcription procedure of the mixture consisted of incubating 20 μL of the solution at 46°C for 20 min, then heating to 95°C for 1 min and finally rapidly cooling to 4°C. Quantitative real‐time PCR reactions were performed using Stratagene Mx3005P (Aligent Technologies) with SsoAdvanced Universal SYBER Green Supermix (BIO‐RAD). Specific primers for CacyBP/SIP (*CACYBP*), ERK1/2 (*MAPK3, MAPK1*), p38 (*MAPK14*) and GAPDH (*GAPDH*) were designed by BIORAD Company. The housekeeping gene GAPDH (GAPDH) was used as a reference gene for quantification. To determine the amounts of levels of test genes expression, standard curves were constructed for each gene separately with serially diluted PCR products. PCR products were obtained by cDNA amplification using specific primers as follows: *CACYBP* (qHsaCED0043669, BIO‐RAD), *MAPK3* (qHsaCID0010939, BIO‐RAD), *MAPK1* (qHsaCED0042738, BIO‐RAD), *MAPK14* (qHsaCED0043417, BIO‐RAD), and *GAPDH* (qHsaCED0038674, BIO‐RAD). QRT‐PCR was carried out in duplicates in a final volume of 10 μL under the following conditions: 2 min polymerase activation at 95°C, 5 s denaturation at 95°C, 30 s annealing at 60°C for 40 cycles. PCR reactions were checked, including no‐RT‐controls, omitting of templates, and melting curve to ensure only one product was amplified. The relative quantification of gene expression was determined by comparing Ct values using the ∆∆Ct method. All results were normalized to GAPDH.

### 2.3 Statistical Analysis

All data obtained from the conducted experiments were statistically analyzed using the Statistica software package (Version 13.3). One‐way ANOVA was used to compare differences between groups. Prior to applying ANOVA, the normality of data distribution was assessed using the Shapiro–Wilk test. Homogeneity of variances was verified, and only in cases where this assumption was met, Fisher's Least Significant Difference (LSD) test was used for post hoc analysis. For RT‐qPCR data, Tukey's Honest Significant Difference (HSD) test was applied.

We acknowledge that in some cases, the control and experimental groups may not be entirely independent, which could affect the assumptions of ANOVA. Therefore, we carefully evaluated the data structure, and where appropriate, alternative statistical approaches are considered to account for potential dependencies. Statistical significance was set at *p* < 0.05.

## 3 Results

**FIGURE 1:**
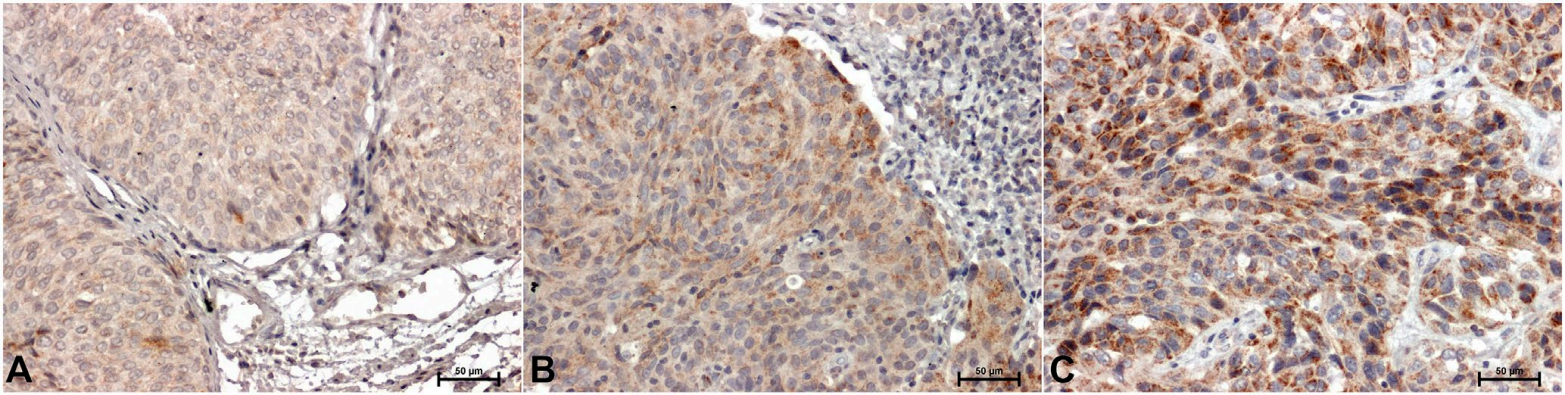
CacyBP/SIP immunodetection in bladder tissue: (A) adjacent normal bladder tissue, (B) low‐grade papillary urothelial carcinoma, (C) high‐grade papillary urothelial carcinoma. ×200. (Olympus BX43 light microscope (Olympus 114 Corp.) with an Olympus DP12 digital camera. PhotoScape 3.7).

**FIGURE 2:**
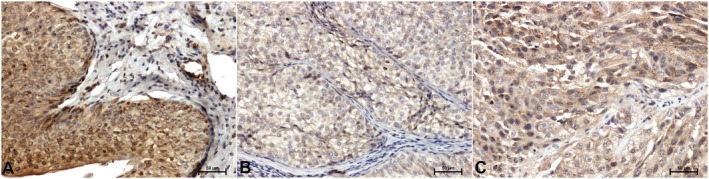
Immunodetection of ERK1/2 in bladder tissue: (A) adjacent normal bladder tissue, (B) low‐grade papillary urothelial carcinoma, (C) high‐grade papillary urothelial carcinoma, ×200 (Olympus BX43 light microscope (Olympus 114 Corp.) with an Olympus DP12 digital camera. PhotoScape 3.7).

**FIGURE 3:**
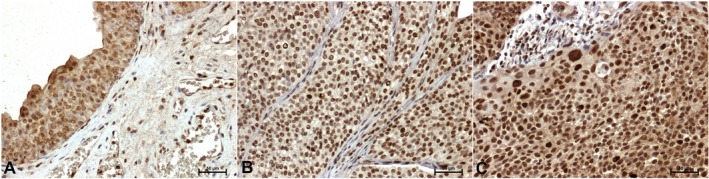
Immunodetection of p38 in bladder tissue: (A) adjacent normal bladder tissue, (B) low‐grade papillary urothelial carcinoma, (C) high‐grade papillary urothelial carcinoma. ×200 (Olympus BX43 light microscope (Olympus 114 Corp.) with an Olympus DP12 digital camera. PhotoScape 3.7).

In the material tested from all patients, immunohistochemical reactions showing CacyBP/SIP, ERK1/2, and p38 were positive. Representative immunostaining of the samples is shown in Figures 1, 2, 3. However, the intensity of the immunoreaction of individual antibodies between adjacent normal bladder tissue material and the tumor tissues was different. Weak, mainly cytoplasmic CacyBP/SIP staining was observed in non‐tumor samples (Figure 1A), while in tumor cells, the reaction was intense (in the cytoplasm) in low‐grade cancer (Figure 1B) or very intense, mainly cytoplasmic, in high‐grade papillary urothelial carcinoma (Figure 1C).

Significantly stronger ERK1/2 immunoreactivity was shown in adjacent normal bladder tissue (Figure 2A) compared to cancer (Figure 2B,C). A particularly strong attenuation of ERK1/2 immunoreactivity compared to controls is observed in low‐grade cancers (Figure 2B). The ERK1/2 immunostaining was primarily observed in the cytoplasm of cells (Figure 2).

Compared with the control (Figure 3A), p38 immunoreactivity was stronger in tumor tissues (Figure 3B,C). The p38 antibody gave the strongest result in the nuclei of high‐grade cancer cells (Figure 3C).

The results of densimetric studies confirmed visual differences in intensity of immunohistochemical reactions against CacyBP/SIP, ERK1/2, and p38 in control tissue and in low‐grade and high‐grade papillary urothelial carcinoma (Table 2).

**TABLE 2:** Intensity of immunoreaction determining CacyBP/SIP, ERK1/2, and p38 in control tissue and in low‐grade and high‐grade papillary urothelial carcinoma (mean ± SE).

	Intensity of immunohistochemical reaction scale from 0 (white pixel) to 255 (black pixel)		
	Control	LGPUC	HGPUC
CacyBP/SIP	71.14 ± 1.37	108.87 ± 1.59*	145.76 ± 1.21*
ERK1/2	120.04 ± 1.93	91.28 ± 1.16*	121.21 ± 1.08
p38	96.81 ± 1.14	143.62 ± 1.11*	159.79 ± 1.22*

* *p* < 0.05 tumor versus control.

**FIGURE 4:**
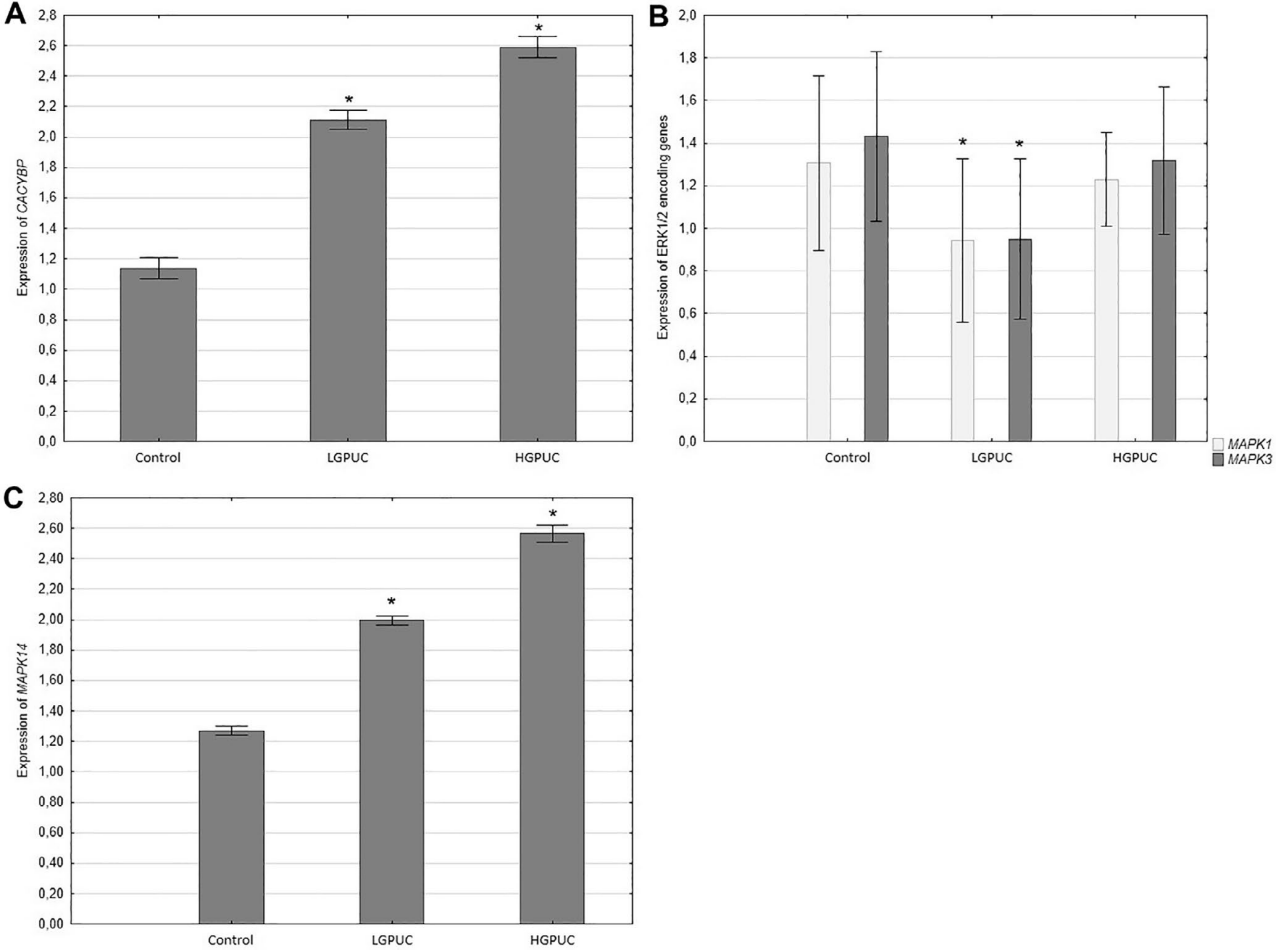
Expression of *CACYBP* genes encoding CacyBP/SIP (A), *MAPK1* and *MAPK3* encoding ERK1/2 (B) and *MAPK14* encoding p38 (C) (mean ± SE) *p < 0.05 tumor versus control (Statistica (Version 13.3), PhotoScape 3.7).

RT‐PCR analysis showed a significant increase in CACYBP and MAPK14 gene expression in tumor tissues (especially in high‐grade urothelial carcinoma) compared to adjacent normal bladder tissue (Figure 4A,C). In contrast, tumor expression of genes (MAPK1, MAPK3) encoding ERK1/2 was decreased, especially in low‐grade tumors, compared to adjacent normal bladder tissue (Figure 4B).

## 4 Discussion

Urothelial cancer of the urinary bladder is the most common malignant tumor of the urinary system. Low‐grade papillary urothelial neoplasms are characterized by mild cytoplasmic atypia and low mitotic potential. Despite these benign parameters, these tumors sometimes develop into high‐grade cancer with an aggressive clinical course and death from unknown causes [24, 25]. High‐grade urothelial carcinoma is characterized by high cytological atypia, a high number of mitoses, and high invasiveness. The main problem in the treatment of this cancer is chemotherapy resistance, and the underlying molecular mechanisms are still unclear [1, 2, 3, 4, 5].

More and more studies show that the CacyBP/SIP protein plays an important role in the initiation, development, invasion, and metastasis of various cancers [18, 26, 27, 28, 29]. The presence of the CacyBP/SIP protein has been found in all types of cancer studied so far [18, 28, 29]. However, the localization, immunoreactivity, and expression level of the gene encoding CacyBP/SIP differed depending on the organ type and tumor stage. For example, in the case of cancer of the stomach, colon, nasopharynx, osteosarcoma, and melanoma, elevated levels of CacyBP/SIP have been demonstrated in relation to adjacent normal bladder tissue [18, 26, 27, 28, 29, 30]. The analysis of literature data and the results of own research indicate that CacyBP/SIP may be involved in various molecular mechanisms in various cancers and perform a complex biological function.

In our previous studies, we demonstrated significantly increased CacyBP/SIP expression in clear cell renal cell carcinoma [31]. In contrast, Ghosh et al. [28] reported lower expression of CacyBP/SIP in renal cancer cell lines and tissues.

To obtain reliable results, we aimed to collect material from a homogeneous group of patients. The number of cases of papillary urothelial carcinoma we have examined is small, but they are homogeneous, which allows us to draw conclusions. Our studies showed a varied increase in CacyBP/SIP expression in bladder cancer tissues, depending on the degree of malignancy. In an in vitro study, Zheng and Chen [32] also showed increased levels of CacyBP/SIP expression in bladder cancer cell lines (T24, UMUC3, BIU‐87, and 5637) compared to normal urothelial cells (SVHUC‐1). The authors of these studies suggest that CacyBP is an important oncogene contributing to the malignant behavior of bladder cancer cells. It turns out that this study on cancer cells is the only one; no other studies on the expression of CacyBP/SIP in bladder cancer have been found in the available literature.

Recent studies indicate that the role of CacyBP/SIP in cancer may be related to the influence on signaling pathways involving MAP kinases and/or its participation in the cellular response to oxidative stress. Moreover, research indicates the participation of oxidative stress in the regulation of MAPK signaling pathways and their involvement in both the initiation and progression of carcinogenesis processes [33].

The essential role of ERK1/2 in the regulation of the epithelial–mesenchymal transition (EMT) is well documented [34, 35]. Selective inhibition of ERK1/2 has been considered a potential cancer treatment strategy that not only effectively blocks the MAPK pathway but also overcomes drug resistance caused by mutations in the RAS, RAF, and MEK genes [36].

In this study, we demonstrated a decrease in ERK1/2 expression in bladder cancer tissues, most notably in low‐grade papillary urothelial carcinoma compared to controls. The results of our research contrast with those of Lin et al. [37], who found significantly higher ERK1/2 expression in epidermal tumors than in non‐cancerous epithelium. Given the well‐documented role of ERK1/2 in intracellular signaling and its involvement in various cancers, our results provide new evidence for its important role also in bladder cancer. Reduced expression of ERK1/2 in cancer cells, especially in low‐grade cancer, may result from disturbances in the biological processes of cancer cells and the biosynthesis of this kinase or weakening of its activity, for example, as a result of dephosphorylation by CacyBP/SIP. On the other hand, the appearance of ERK1/2 in the nuclei of high‐grade bladder cancer cells may indicate the involvement of this kinase in processes related to transcription and protein coding.

Studies have shown that p38 kinase is involved in angiogenesis, cell proliferation, inflammation, and the production of immunomodulatory cytokines [15]. Increased survival of cancer cells is associated with chronic inflammation. MAP kinase, through its ability to regulate the expression of important inflammatory mediators, may promote oncogenesis via cellular mechanisms and interactions with the tumor microenvironment [38].

In this study, we found that both p38 mRNA and protein levels are increased in papillary urothelial carcinoma compared with adjacent, noncancerous bladder tissue. We also showed that p38 mRNA and protein expression was highest in high‐grade lesions, while the difference in expression between low‐grade carcinoma and adjacent normal bladder urothelium was less pronounced.

An elevated level of p‐p38 positively correlated with the degree of malignancy has been reported in breast, lung, and thyroid cancer [38]. The p38 protein functions in a cell type and functional state‐specific manner to integrate signals that influence proliferation, differentiation, survival, and migration.

Reactive oxygen species, by modulating the activity of receptors, transcription factors, and protein kinases, activate oncogenic cell signaling pathways. An example is ERK kinases belonging to the MAPK family. The active (phosphorylated) form of ERK activates transcription factors that regulate the expression of genes related to cell growth and division [39].

Differential expression of the CACYBP/SIP protein in pathological processes related to oncogenesis indicates that CacyBP/SIP, through interactions with various proteins, may participate in many processes and signaling pathways and act as an oncogene or tumor suppressor. Signaling pathways involving ERK1/2 and p38 kinases play a very important role in maintaining tissue homeostasis, in the processes of cell survival and differentiation, and consequently in cancer. Based on available literature data and the analysis of our own research results, it can be assumed that the expression of CacyBP/SIP depends on the type and degree of histological malignancy of the tumor.

In this paper, we present for the first time the results of studies indicating a significant, tumor grade‐dependent increase in the expression of the CacyBP/SIP protein in papillary urothelial carcinoma and the potential function of this protein in relation to ERK1/2 and p38 kinases. The results clearly suggest that the CacyBP/SIP protein has the potential to control carcinogenesis by regulating ERK1/2 and p38 kinases involved in the pathways associated with papillary bladder cancer‐related pathways.

Further studies are needed to better understand how CacyBP/SIPs participates in cancer development by regulating MAP kinase pathways. Perhaps regulation of specific components of this elements signaling pathway represents a promising therapeutic strategy. However, much remains to be clarified, and our results justify continuing this line of research.

A limitation of our pilot study that should be taken into consideration is the relatively small number of participants in both the bladder cancer group and the control group. While the findings indicating novel molecular pathways involved in cancer development—particularly in chemotherapy‐resistant cases—are potentially promising, they should be interpreted with caution. Further research involving larger cohorts and more detailed molecular techniques is necessary to accurately assess the contribution of the studied parameters to the initiation and progression of bladder cancer.

## 5 Conclusion

These findings offer novel perspectives on the molecular pathways implicated in bladder cancer, potentially paving the way for the identification of promising therapeutic targets in the context of chemotherapy‐resistant bladder cancer. However, understanding what determines the function (potentially as a phosphatase) of the CacyBP/SIP protein in regulating MAPK signaling pathways, which may be either oncogenic or sometimes inhibit tumor growth through mechanisms that have not yet been elucidated, requires further research. Understanding these pathways may be important in defining effective treatment strategies for urological cancers.

Further studies on a larger number of cases and the use of additional molecular methods are necessary to precisely assess the contribution of the studied parameters to the emergence and development of the examined tumor, which may constitute a novel molecular target for clinical trials in the future.

## Author Contributions

**Natalia Domian:** conceptualization (lead), data curation (lead), formal analysis (equal), investigation (equal), methodology (equal), resources (equal), validation (equal), visualization (equal), writing – original draft (lead). **Grzegorz Młynarczyk:** data curation (equal), investigation (supporting), resources (supporting). **Irena Kasacka:** conceptualization (equal), data curation (equal), investigation (equal), methodology (equal), resources (equal), supervision (lead), writing – review and editing (lead).

## Funding

This work was supported by Medical University of Białystok (Medical University of Bialystok) B.SUB.23.422.

## Disclosure

Declaration of Figures' Authenticity: All figures submitted have been created by the authors, who confirm that the images are original with no duplication and have not been previously published in whole or in part.

## Ethics Statement

Informed consent was obtained from all subjects involved in the study. The study was approved by the Bioethics Committee of the Medical University of Bialystok. The code for our study is APK.002.368.2023. All research was performed in accordance with relevant guidelines/regulations. Research involving human research participants has been performed in accordance with the Declaration of Helsinki.

## Conflicts of Interest

The authors declare no conflicts of interest.

## Data Availability

The data that support the findings of this study are available on request from the corresponding author. The data are not publicly available due to privacy or ethical restrictions.
